# Cerebrospinal fluid neurofilament dynamic profiles predict cognitive progression in individuals with *de novo* Parkinson’s disease

**DOI:** 10.3389/fnagi.2022.1061096

**Published:** 2022-12-16

**Authors:** Ze-Hu Sheng, Ling-Zhi Ma, Jia-Yao Liu, Ya-Nan Ou, Bing Zhao, Ya-Hui Ma, Lan Tan

**Affiliations:** Department of Neurology, Qingdao Municipal Hospital, Qingdao University, Qingdao, China

**Keywords:** cerebrospinal fluid, neurofilament light, Parkinson’s progression markers initiative, cognitive decline, Parkinson’s disease

## Abstract

**Background:**

Neurofilament light chain protein (NfL) in cerebrospinal fluid (CSF) reflects the severity of neurodegeneration, with its altered concentrations discovered in Parkinson’s disease (PD) and Parkinson’s disease dementia (PD-D).

**Objective:**

To determine whether CSF NfL, a promising biomarker of neuronal/axonal damage, can be used to monitor cognitive progression in *de novo* Parkinson’s disease and predict future cognitive decline.

**Methods:**

A total of 259 people were recruited in this study, including 85 healthy controls (HC) and 174 neonatal PD patients from the Parkinson’s Progression Markers Initiative (PPMI). Multiple linear regression and linear mixed effects models were used to examine the associations of baseline/longitudinal CSF NfL with cognitive decline and other CSF biomarkers. Kaplan–Meier analysis and log-rank test were used to compare the cumulative probability risk of cognition progression during the follow-up. Multivariate cox regression was used to detect cognitive progression in *de novo* PD.

**Results:**

We found PD patients with mild cognitive impairment (PD-MCI) was higher than with normal cognition (PD-NC) in terms of CSF NfL baseline levels (*p* = 0.003) and longitudinal increase rate (*p* = 0.034). Both baseline CSF NfL and its rate of change predicted measurable cognitive decline in *de novo* PD (MoCA, *β* = −0.010, *p* = 0.011; *β* = −0.0002, *p* < 0.001, respectively). The predictive effects in *de novo* PD patients aged >65, male, ill-educated (<13 years) and without carrying Apolipoprotein E *ε4* (*APOE ε4*) seemed to be more obvious and reflected in more domains investigated. We also observed that CSF NfL levels predicted progression in *de novo* PD patients with different cognitive diagnosis and amyloid status. After an average follow-up of 6.66 ± 2.54 years, higher concentration above the median of baseline CSF NfL was associated with a future high risk of PD with dementia (adjusted HR 2.82, 95% CI: 1.11–7.20, *p* = 0.030).

**Conclusion:**

Our results indicated that CSF NfL is a promising prognostic predictor of PD, and its concentration and dynamics can monitor the severity and progression of cognitive decline in *de novo* PD patients.

## Introduction

Parkinson’s disease (PD) secondary to basal ganglia dysfunction is a complex ailment in terms of epidemiology, pathology, genetics, clinical expression, and therapy (Kalia and Lang, 2015). Due to an aging population, the number individuals afflicted with this second most common neurodegenerative disease is anticipated to double in 20 years (Mehta and Adler, 2016). Approximately 15–40% of PD patients with mild cognitive impairment (PD-MCI) can be offered a diagnosis when motor symptoms are observed. In patients with PD, cognitive decline has been recognized as a factor that further worsens a patient’s prognosis, as compared to other disease-related non-motor symptoms (Mollenhauer et al., 2014). Meanwhile, the incidence of PD with dementia (PD-D) ranges within 24 to 31%, and rises to 80% in a 20-year duration (Aarsland et al., 2005b; Song et al., 2011; Leaver and Poston, 2015). The lack of great approaches to predict the time frame of disease progression possibly contributes to poor prognosis and serious economic burden to PD patients (Findley et al., 2003). Therefore, it is of utmost importance to investigate whether certain biomarkers in cerebrospinal fluid (CSF) can predict or monitor the extent of cognitive deterioration in PD patients (Hall et al., 2015).

Neuroaxonal degeneration is a significant pathological mechanism that generates permanent disability in neurological disorders (Ng et al., 2020). Neurofilament light (NfL) is an enriched cytoskeletal protein exclusively expressed by central and peripheral neurons; it is also released to CSF after axonal injury and neurodegeneration (Bridel et al., 2019). CSF NfL provides a sensitive measurement for the identification of neuroaxonal damage, as well as the extent of this damage (Khalil et al., 2018). Recent studies have found that CSF NfL concentration is significantly elevated in PD-MCI and PD-D patients in comparison to healthy controls (HC; Hall et al., 2012; Lerche et al., 2020). Nonetheless, whether this increase in concentration presents in early PD patients with cognitive decline remains uncertain. Longitudinal data of CSF biomarkers in PD population is rare, and comprehensive longitudinal models for cognitive prediction are deficient in past research studies. It is essential to critically discuss and further explore the predictive value of CSF NfL to validate whether this effect exists in independent PD cohorts.

Considering CSF NfL as a promising biomarker in neurodegenerative diseases, we hypothesize that its concentration and dynamic analysis may possibly predict cognitive progression and detect related pathological changes in *de novo* PD patients. In the present research, we investigated the associations of CSF NfL with cognitive progression on different aspects and CSF biomarkers through cross-sectional and longitudinal analyses based on different diagnostic groups of studied population from the Parkinson’s Progression Markers Initiative (PPMI) database (2011).

## Materials and methods

### Participants from PPMI

We obtained the data from the PPMI database on February 27, 2022.^1^ The PPMI is an international multi-center, ongoing, observational, longitudinal study that aims to identify biomarkers of PD progression and thus accelerate clinical trials and development of therapies (Marek et al., 2011). PD population included must be ≥30 years when diagnosed; have an asymmetric resting tremor or asymmetric bradykinesia or two of bradykinesia, resting tremor and rigidity within two years after diagnosis of PD (Hoehn-Yahr Stage I or II at Baseline; Lewis et al., 2005; Selikhova et al., 2009; Eggers et al., 2012); be untreated for PD especially not using drugs that might impact dopamine transporter imaging and CSF composition; have no dementia as determined by the investigator. Longitudinal review of the diagnosis conducted by researchers aims to prevent misdiagnosis. PD subjects should be excluded from follow-up if suspected as PSP or MSA. Pregnant, lactating women or planning pregnancy during the course are also not included. HC could be enrolled if they had no significant neurologic disorder, Montreal Cognitive Assessment (MoCA) > 26 and first-degree relatives without PD (2011). Both PD subjects and HC were assessed in clinical and CSF biomarkers study. In our research, participants were obliged to have baseline CSF NfL data and at least one more additional monitoring CSF NfL in later visit. As a result, CSF NfL concentration was measured at baseline (BL), 0.5, 1, 2, 3 and 4 years, and 1,166 data were collected altogether. After the first CSF NfL collected, Longitudinal follow-up analyzed of other 4 CSF biomarkers and 6 clinical assessments (Supplementary Table 1).

### Analyses of CSF biomarkers

Details about collection and analyses for CSF biomarkers can be read in the PPMI biologics manual (Kang et al., 2013). Roche NTK was used to quantify CSF NfL on a cobas e411 analyzer at Covance Greenfield laboratories (Translational Biomarker Solutions, Indiana; Bartl et al., 2021). Likewise, above-mentioned sandwich immunoassay was used to measure CSF total α-synuclein (α-syn) by Bio-Legend (San Diego, CA; Kang et al., 2016). CSF amyloid-β_42_ (Aβ_42_), total tau (T-tau), and phosphorylated tau (P-tau) were measured by electrochemiluminescence (ECL) method on a completely automated cobas e601 analyzer (Shaw et al., 2018).

### Clinical assessment measures

As reported elsewhere, various cognitive function evaluations have been performed for each clinical visit in the PPMI study (Kang et al., 2013). Since the system of cognitive disorder in PD is highly variable in its scope and intensity involving various filed, only using MoCA or MMSE as a screening instrument is not comprehensive enough (Caviness, 2007). We selected the following 6 cognitive function tests in different areas: MoCA for global cognition, Hopkins Verbal Learning Test for episodic memory, Wechsler Memory Scale third edition Letter-Number Sequencing test (LNS) for verbal working memory, Symbol Digit Modalities Test (SDMT) for processing speed-attention, Semantic Fluency Test for language, Benton Judgment of Line Orientation Score (BJLO) for visuospatial function (Kang et al., 2013; Schrag et al., 2017). Considering MoCA is a suitably accurate, brief instrument with adequate specificity and sensitivity of psychometric properties when screening degrees of cognition in PD (Dalrymple-Alford et al., 2010). In addition to evidence of detecting PD-MCI or PD-D (Hendershott et al., 2017), MoCA has been verified in several language superior to the standardized MMSE in several PD cohort studies (Chou et al., 2010). Cognitive status of study population was defined according to MDS level I guideline: MoCA score was <22 defined as PD-D, PD-MCI if MoCA score was 22–26, finally population scored MoCA >26 defined as PD patients with normal cognition (PD-NC; Hoops et al., 2009; Litvan et al., 2012; Chahine et al., 2016).

### Statistical analyses

We used the R (version 4.1.0) to perform statistical analyses. Comparisons of demographic, clinical assessments and CSF biomarkers were made between HC, PD-NC and PD-MCI. Chi-square tests with continuity correction were used for categorical variables; Student’s *t*-tests or Wilcoxon rank-sum tests were used for continuous variables. Shapiro–Wilk tests were used to check normal distribution and Bartlett tests of homogeneity were used for variances. Differences in characteristics between groups were analyzed using the one-way ANOVA or Kruskal-Wallis with Dunn *post hoc* tests. CSF NfL levels did not distribute normally (*p* < 0.05). To be proximity of normal distribution, we used log10-transformed method (Supplementary Figure 1). Then other needed indicators in our study were applied with same normalization method. We excluded 7 participants in reference to outliers which were defined as mean value ±3 SD to eliminate the influence of extreme values. Multiple linear regression model was used to examine the association of CSF NfL baseline concentration with data of other CSF biomarkers and cognitive assessments. Meanwhile, multiple linear mixed-effects (LME) model was used to calculate random slope and intercepts terms modeling CSF NfL longitudinal rate. Prediction of other cognitive measurements using baseline concentration or change rate of CSF NfL was also tested by the multiple LME model. We evaluated models with median of NfL baseline levels or longitudinal change rate. Low CSF Aβ and elevated tau (T-tau and P-tau) levels had been shown to predict progression of cognitive decline specifically in cognitive impairment and Alzheimer’s disease (AD; Diniz et al., 2008). We selected the transforming formula (Shaw et al., 2019) which has been proved credible and used in previous studies (Kang et al., 2016; Irwin et al., 2020), to shift Elecsys values to equivalent values of AlzBio3 [*x* = (CSF Aβ_42_ + 251.55)/3.74] to reduce the bias caused by difference of CSF Aβ_42_ levels between PPMI and AD cohorts (Stewart et al., 2019), and considered cut off value <250 pg/ml of corresponding AlzBio3 (Shaw et al., 2018) to distinguish amyloid positivity (A+) or amyloid negativity (A−). Kaplan–Meier curves were used to compare the cumulative probability risk of cognitive progression in the follow-up among different groups. Besides multivariate cox regression model was used to analysis the association between CSF NfL and the occurrence of conversion to the PD-D during the follow-up. We then added ‘medical comorbidities’ as a covariate in multiple linear regression, linear mixed effects and multivariate cox regression models to conduct sensitivity analyses.

The covariates of all regression and LME analyses were age, gender, educated years, Apolipoprotein E *ε4* (*APOE ε4*) carrier status, and disease duration, and the significance threshold was set at *p* < 0.05. All *de novo* PD patients were classified by age (<56, 56–65, >65), gender, educated years (≥13, <13), *APOE ε4* carrier status to investigate the associations between CSF NfL and various cognitive indicators.

## Results

### Study participants

Included participants consisted of cognitively intact HC (n = 85), PD-NC (n = 116) and PD-MCI (*n* = 58; Table 1). There were no significant differences in sex, educated years and *APOE ε4* allele status between HC, PD-NC and PD-MCI, which was closely similar to the previous studies of PPMI cohort (Kang et al., 2016). As expected, cognitive performance differed in three populations (MoCA, *p* < 0.001; HVLT Total Recall, *p* = 0.007; HVLT Delayed Recall, *p* = 0.002; HVLT Recognition Discrimination, *p* = 0.011; Semantic Fluency Test, *p* < 0.001). Age and CSF NfL levels were significantly different between three groups, meanwhile age positively correlated with CSF NfL in each group (HC: *β* = 2.2983, *p* < 0.001; PD-NC: *β* = 2.3492, *p* < 0.001; PD-MCI: *β* = 3.5410, *p* < 0.001; Supplementary Figure 2) which was also consistent with reported study (Lerche et al., 2020; Fagan et al., 2021).

**Table 1:** Baseline clinical information and NfL concentration of participants in this study.

Characteristics	HC (*n* = 85)	PD-NC (*n* = 116)	PD-MCI (*n* = 58)	*P*
Age (y)	62.31 ± 10.14	59.00 ± 9.69	63.75 ± 9.72	**0.005**
Gender (Female/Male)	28/57	38/78	20/38	0.972
Education (y)	16.14 ± 2.55	16.29 ± 2.44	15.76 ± 2.68	0.308
*APOE ε4* carriers (%)	22 (25.9)	33 ± 28.4	15 ± 25.9	0.712
Aβ_42_ (pg/mL)	1019.89 ± 439.97	931.02 ± 368.54	909.22 ± 393.35	0.322
T-tau (pg/mL)	190.66 ± 67.39	169.48 ± 48.72	173.95 ± 60.35	0.113
P-tau (pg/mL)	16.82 ± 6.63	14.52 ± 4.67	15.02 ± 5.89	0.058
α-syn (pg/mL)	1649.29 ± 646.94	1500.39 ± 559.36	1580.20 ± 671.16	0.271
NfL (pg/mL)	97.94 ± 43.29	91.33 ± 46.07	119.89 ± 65.35	**0.009**
Montreal Cognitive Assessment	28.13 ± 1.08	28.26 ± 1.03	24.71 ± 1.39	**<0.001**
HVLT Total Recall	48.60 ± 10.71	47.84 ± 9.94	43.29 ± 9.39	**0.007**
HVLT Delayed Recall	47.45 ± 12.55	47.49 ± 10.05	41.67 ± 10.77	**0.002**
HVLT Retention	48.12 ± 12.68	48.35 ± 9.88	45.71 ± 13.58	0.122
HVLT Recognition Discrimination	47.14 ± 12.43	47.41 ± 10.39	42.31 ± 12.16	**0.011**
Benton Judgment of Line Orientation Score	12.19 ± 2.96	12.84 ± 2.50	11.97 ± 3.11	0.170
Letter Number Sequencing	11.76 ± 2.93	11.80 ± 2.47	10.81 ± 2.49	0.072
Semantic Fluency Test	51.25 ± 10.36	53.38 ± 9.14	47.07 ± 9.43	**<0.001**
Symbol Digit Modality Test	48.55 ± 10.86	46.18 ± 8.40	44.38 ± 9.48	0.079

NfL, Neurofilament light; HC, Healthy controls; PD, Parkinson’s disease; PD-NC, PD patients with normal cognition; PD-MCI, PD patients with mild cognitive impairment; APOE, Apolipoprotein E; Aβ_42_, Amyloid-β_42_; T-tau, Total tau; P-tau, Phosphorylated tau; α-syn, α-synuclein; HVLT, Hopkins Verbal Learning Test; values are mean ± standard deviation (SD), or n or n (% of the group).

### Cross-sectional analyses of CSF NfL baseline concentration

**Figure 1:**
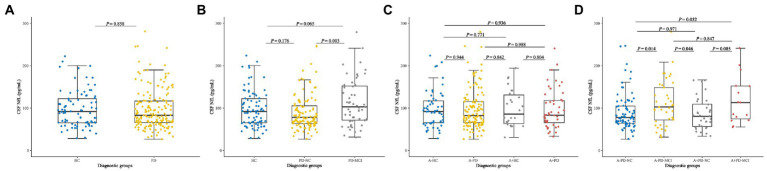
Baseline CSF NfL concentration of **(A)** Healthy controls and patients with *de novo* PD. **(B)** HC and *de novo* PD patients with different cognitive status. **(C)** HC and *de novo* PD patients in different amyloid status. **(D)** HC and de novo PD patients with different cognitive status in different amyloid status. CSF, cerebrospinal fluid; NfL, neurofilament light; HC, Healthy controls; PD, Parkinson’s disease; PD-NC, PD patients with normal cognition; PD-MCI, PD patients with mild cognitive impairment; A, Amyloid.

No significant differences of baseline CSF NfL levels were found between *de novo* PD patients and HC (Figure 1A). NfL concentration was higher in PD-MCI (119.89 pg/ml) than PD-NC (91.33 pg/ml; *p* = 0.003; Figure 1B). Considering the acknowledged cut-point in AD, we cross-sectionally investigated HC, PD-NC and PD-MCI at baseline with presumed amyloid-positivity (Yarnall et al., 2014). Relative of low CSF Aβ_42_ in pathology of amyloidosis (A+) in PD group (28.16%) and HC group (30.59%) had no differences in CSF NfL baseline concentration between A + and A-groups (Figure 1C). However, CSF NfL levels were significantly higher in the A-PD-MCI (119.90 pg/ml) compared to A-PD-NC (90.46 pg/ml; *p* = 0.014). We also found difference in NfL concentration between A-PD-MCI (119.90 pg/ml) and A + PD-NC (93.44 pg/ml; *p* = 0.046; Figure 1D).

After considered and adjusted age, gender, educated years, *APOE ε4* carrying status, and PD duration, we found the significant correlations between CSF NfL levels and cognition severity (MoCA, *β* = −0.031, *p* = 0.045) in *de novo* PD patients.

### Prediction and CSF NfL baseline concentration in *de novo* PD patients

**Figure 2:**
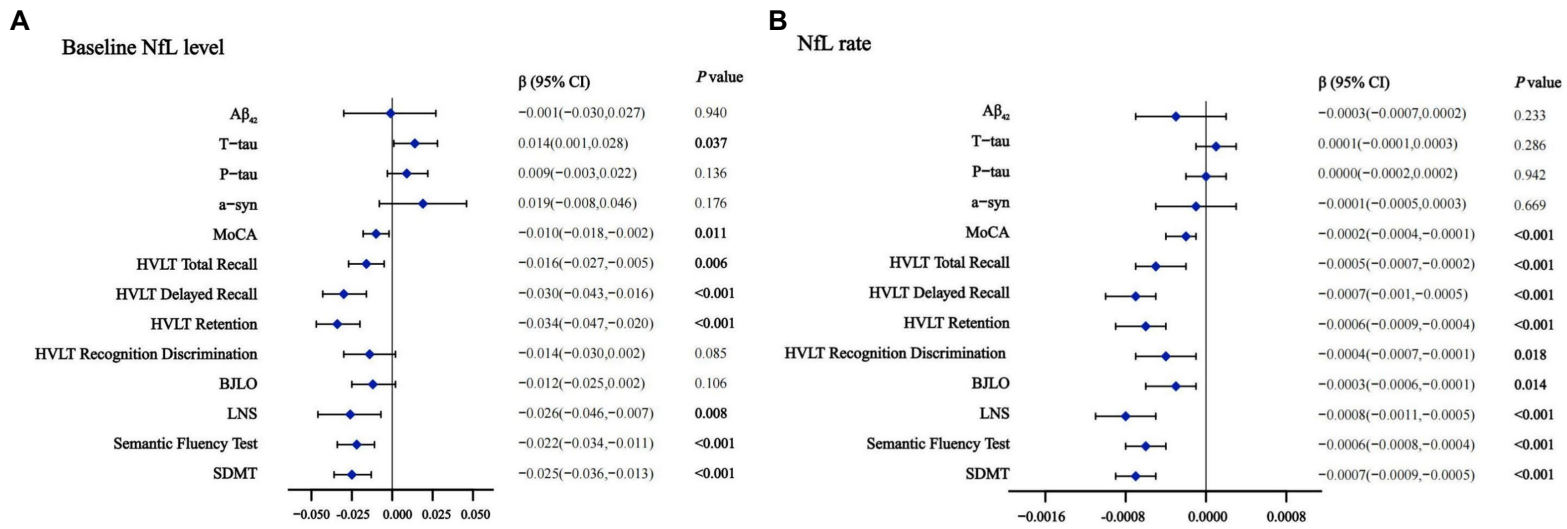
Effects of baseline NfL **(A)** and NfL accumulation rate **(B)** on CSF biomarkers and cognition measurements in linear mixed-effects analysis among PD participants. Adjusted for age, gender, educated years, *APOE ε4* carrier status and duration. CSF, cerebrospinal fluid; NfL, neurofilament light; PD, Parkinson’s disease; Aβ_42_, Amyloid-β_42_; T-tau, Total tau; P-tau, Phosphorylated tau; α-syn, α-synuclein; MoCA, Montreal Cognitive Assessment; HVLT, Hopkins Verbal Learning Test; BJLO, Benton Judgment of Line Orientation Score; LNS, Letter Number Sequencing; SDMT, Symbol Digit Modality Test.

To exploratorily analyze the prediction using CSF NfL in early PD cognitive decline according to reported discovery (Lerche et al., 2020; Oosterveld et al., 2020; Aamodt et al., 2021), we hypothesize that upregulation of CSF NfL will associate with cognitive progression in several domains. In PD population, higher CSF NfL baseline concentration predicted faster decline in global cognition (MOCA, *β* = −0.010, *p =* 0.011), episodic memory (HVLT Total Recall, *β* = −0.016, *p =* 0.006; HVLT Delayed Recall, *β* = −0.030, *p <* 0.001; HVLT Retention, *β* = −0.034, *p <* 0.001), verbal working memory (LNS, *β* = −0.026, *p =* 0.008), language (Semantic Fluency Test, *β* = −0.022, *p <* 0.001) and processing speed-attention (SDMT, *β* = −0.025, *p <* 0.001; Figure 2A). Compared with the low NfL Concentration below the median, higher CSF NfL Concentration above the median can predict decline in most of investigated cognitive domains except visuospatial functioning (Supplementary Table 3).

**Figure 3:**
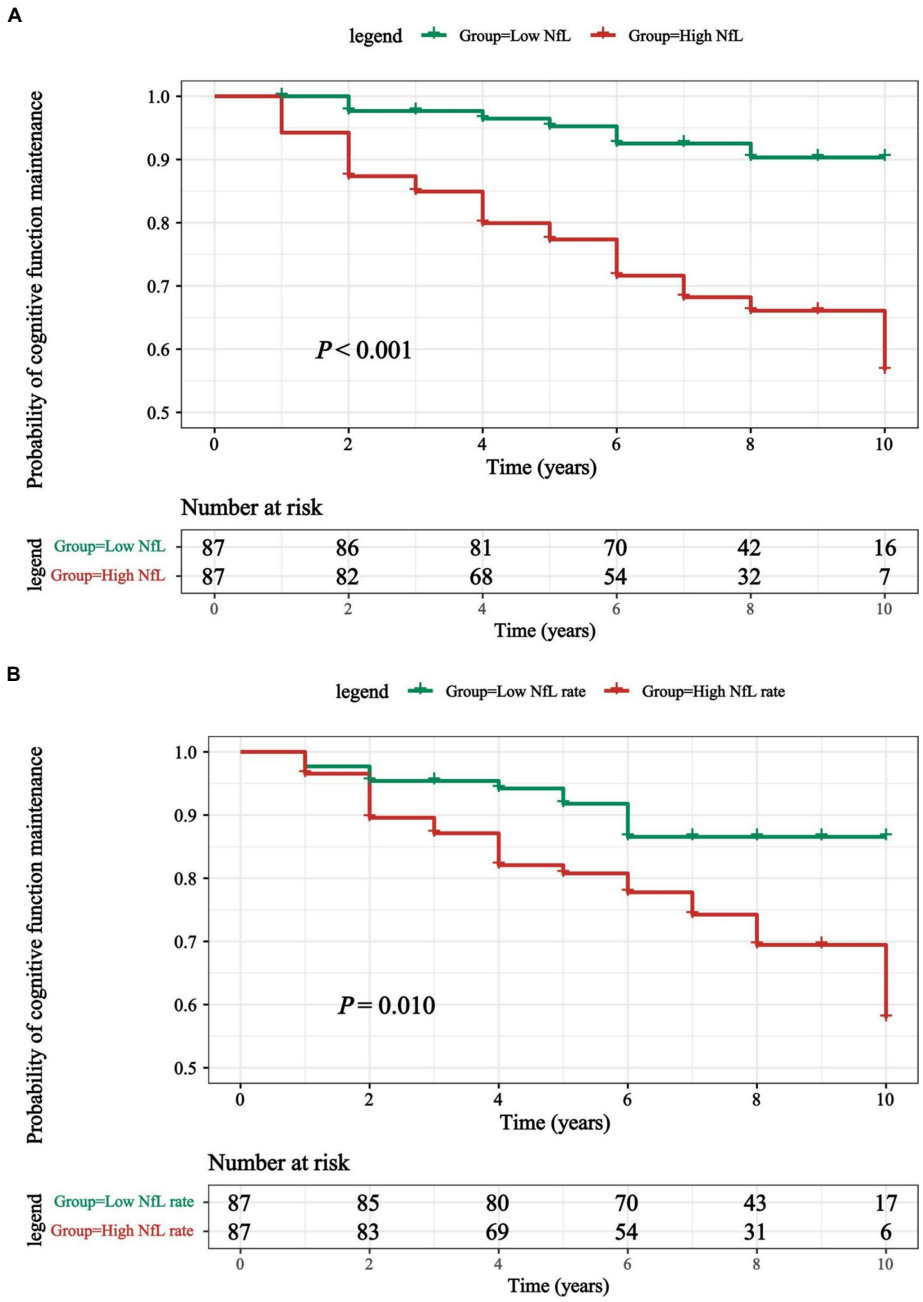
**(A)** Kaplan-Meier curves were used to compare the cumulative probability risk of cognitive progression in the follow-up among participants which were divided into 2 groups by the median of CSF NfL levels. **(B)** Kaplan-Meier curves were used to compare the cumulative probability risk of cognitive progression in the follow-up among participants which were divided into 2 groups by the median of CSF NfL change rate. Kaplan–Meier curve for conversion from *de novo* PD patients converted to PD-D during 9-years follow-up. PD, Parkinson’s disease; PD-D, Parkinson’s disease with dementia; NfL, neurofilament light.

Results of Kaplan–Meier analysis displays a remarkable decline of MoCA score, and log-rank test has statistical significance (*p* < 0.001; Figure 3A). In addition, 34 of 174 PD patients (16.28%) had transition to PD-D during a mean follow-up period of 6.66 ± 2.54 years. Cox proportional-hazards models were used to estimate the conversion risk from PD patients with non-dementia to PD-D (Supplementary Table 4). PD individuals with higher CSF NfL levels above the median added an increased risk of conversion to PD-D compared with the subjects in low CSF NfL concentrations below the median (HR = 2.82, 95% CI: 1.11–7.20, *p* = 0.030).

### CSF NfL baseline concentration and other CSF biomarkers

Cross-sectionally Significant associations were observed between baseline NfL and other CSF biomarkers both in the HC (T-tau, *β* = 0.484, *p* < 0.001; P-tau, *β* = 0.540, *p* < 0.001; α-syn, *β* = 0.506, *p* < 0.001) and PD patients (T-tau, *β* = 0.247, *p* < 0.001; P-tau, *β* = 0.256, *p* < 0.001; α-syn, *β* = 0.192, *p* = 0.013; Aβ42, *β* = 0.182, *p* = 0.027). In PD-NC and PD-MCI group, CSF NfL levels were also significantly associated with CSF T-tau and P-tau too (Supplementary Table 2). Baseline NfL levels also predicted the longitudinal increase of CSF T-tau (*β* = 0.014, *p =* 0.037) by the multiple LME models (Figure 2A). Additionally, the associations were pronounced between higher baseline NfL concentration above the median (T-tau, *β* = 0.007, *p =* 0.013; P-tau, *β* = 0.005, *p =* 0.037; Supplementary Table 3).

### Longitudinal change in CSF NfL

**Figure 4:**
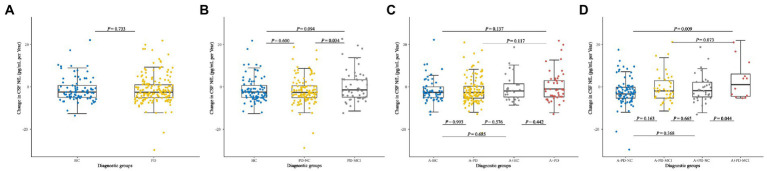
Longitudinal changes in CSF NfL of **(A)** Healthy controls and patients with *de novo* PD. **(B)** HC and *de novo* PD patients with different cognitive status. **(C)** HC and *de novo* PD patients in different amyloid status. **(D)** HC and *de novo* PD patients with different cognitive status in different amyloid status. CSF, cerebrospinal fluid; NfL, neurofilament light; HC, Healthy controls; PD, Parkinson’s disease; PD-NC, PD patients with normal cognition; PD-MCI, PD patients with mild cognitive impairment; A, Amyloid.

The Longitudinal profile of CSF NfL was further examined from baseline in PD population and HC by using LME models. The NfL change rate was slightly above zero in *de novo* PD patients (0.60 pg/ml/a, Figure 4A). CSF NfL concentration increased significantly with higher rate in PD-MCI (5.67 pg/ml/a) than PD-NC (−1.94 pg/ml per year; *p* = 0.034; Figure 4B). There were no significant differences of CSF NfL rate between *de novo* PD patients and HC in different amyloid status (Figure 4C). Finally, A+ PD-MCI (16.02 pg/ml per year) increased faster with highest NfL growth than both A+ PD-NC (−0.86 pg/ml per year; *p* = 0.044) and A-PD-NC (−2.39 pg/ml per year; *p* = 0.009; Figure 4D).

### Prediction and CSF NfL change rate in *de novo* PD patients

Analyses of cognitive decline using CSF NfL change rate demonstrated that 4 years longitudinally increased CSF NfL concentration was correlated with cognitive performance significantly. We observed higher increasing CSF NfL rate predicted faster worsening in global cognition (MOCA, *β* = −0.0002, *p <* 0.001) in episodic memory (HVLT Total Recall, *β* = −0.0005, *p <* 0.001; HVLT Delayed Recall, *β* = −0.0007, *p <* 0.001; HVLT Retention, *β* = −0.0006, *p <* 0.001; HVLT Recognition Discrimination, *β* = −0.0004, *p =* 0.018), visuospatial functioning (BJLO, *β* = −0.0003, *p =* 0.014), verbal working memory (LNS, *β* = −0.0008, *p <* 0.001), language (Semantic Fluency Test, *β* = −0.0006, *p <* 0.001), and processing speed-attention (SDMT, *β* = −0.0007, *p <* 0.001; Figure 2B). Compared with the low NfL rate below the median higher change rate of NfL above the median can predict a decline in most of investigated cognitive domains except visuospatial functioning. No significant associations were observed between longitudinal CSF NfL and other CSF biomarkers (Supplementary Table 3).

Although no correlation between CSF NfL change rate and the risk of PD-D transformation according to Cox proportional-hazards models was found (Supplementary Table 4), the results of Kaplan–Meier analysis and log rank test demonstrated that differences existed comparing two groups divided by median of NfL change rates (*p* = 0.010; Figure 3B).

### Subgroup analyses

In *de novo* PD patients aged >65, male, ill-educated (<13 years) and without carrying *APOE ε4*, we found the predictive effects of CSF NfL baseline concentration and longitudinal rate on cognitive decline appeared to be more remarkable and comprehensive. (Supplementary Tables 5–8). In PD-NC, NfL baseline concentration and longitudinal rate predicted cognitive decline in episodic memory and verbal working memory. In PD-MCI, NfL baseline concentration and longitudinal rate predicted cognitive decline in episodic memory, language and processing speed-attention. (Supplementary Table 9). In A-PD group, predictive effects of NfL baseline levels and longitudinal rate were observed not only on cognitive decline in episodic memory, language and processing speed-attention but also on increase of CSF T-tau and P-tau. In A + PD group, the NfL baseline levels and longitudinal rate showed significant predictive effects on cognitive decline in global cognition, verbal working memory, language and processing speed-attention (Supplementary Table 10).

### Sensitivity analyses

After adjusting for medical comorbidities, the results were consistent with the previous fingings (Supplementary Tables 11–13), consolidating the predictive value of CSF NFL on cognition.

## Discussion

This study involves a well-designed longitudinal cohort of *de novo* PD patients to comprehensively investigate the predictive value of cerebrospinal fluid NfL linked to various indicators of cognitive decline. The analyses demonstrated the following: (1) PD-MCI was significantly higher than PD-NC in terms of CSF NfL baseline concentration and longitudinal change rate; (2) CSF NfL predicted the longitudinal cognitive progression of *de novo* PD patients and successfully marked conversion to cognitive impairment beforehand; and (3) The predictive effects of CSF NfL baseline concentration and the cognitive decline change rate among *de novo* PD male patients aged >65 who were ill-educated and without *APOE ε4* carrier status seemed to be more obvious.

We found cross-sectional correlation between CSF NfL concentration and global cognition in PD patients, which fit a reported study (Lerche et al., 2020). In the PD group, we employed LME models to predict disease time frame and cognitive progression. The results showed that the CSF NfL change rate predicted greater decline in six investigated domains over an average of 6.66 years follow-up. Moreover, baseline NfL concentration can also predict these cognitive domains except for visuospatial functioning. Differing from a previous study that reported an increase in CSF NfL levels with cognitive dysfunction without marking conversion to cognitive impairment (Lerche et al., 2020), a higher CSF NfL concentration above the median added risk of conversion to the PD-D in our study. However, this outcome was consistent with another cohort study (Bäckström et al., 2015), and provides the possibility of CSF NfL dictating predictive values for general early cognitive decline in PD. NfL levels might be impacted by medical comorbidities that are common among elders, complicating its utility as a biomarker. Although multiple sclerosis, neurodegenerative dementia, stroke, amyotrophic lateral sclerosis (Khalil et al., 2018), orthostatic hypotension (Park et al., 2021), hearing disorder (Park et al., 2016; Han et al., 2018), depression and anxiety disorders (Tauil et al., 2021), diabetes (Thota et al., 2022), psoriasis (Nowowiejska et al., 2022), transient ischemic attack (TIA; De Marchis et al., 2018) and sleep disorder (Jennum et al., 2017; Lysen et al., 2020; Targa et al., 2021) might impact NfL levels, we still found consistent results after adjusting for medical comorbidities. CSF NfL can purposefully play the role of a future candidate for CSF biomarkers when cognitive disorder risk is estimated among patients with PD (Hoops et al., 2009).

Substantial existing biological heterogeneity and pathological evidence underlie the clinical features (cognitive impairment) of Parkinson’s disease (Chen-Plotkin et al., 2018). The etiology of PD cognitive disorder is complex with several pathological mechanisms (Aarsland et al., 2017). There are a few cohort studies that systematically investigated longitudinal changes in the CSF biomarkers of PD patients. A previous study confirmed the correlation between CSF NfL concentration, CSF Aβ_42_, and T-tau levels in PD (Aamodt et al., 2021). We further examined the relationship between CSF NfL and AD-related CSF biomarkers. CSF T-tau is presumed to be correlated with cognitive impairment in PD (Ritchie et al., 2017; Schrag et al., 2017). In our research, CSF T-tau and P-tau levels were cross-sectionally correlated with CSF NfL. We found that NfL concentration can predict the increase of CSF T-tau. Results from several autopsy studies demonstrated that limbic and neocortical Lewy body depositions were major determinants of cognitive decline in PD (Braak et al., 2005; Aarsland et al., 2005a). Nevertheless, other studies favor the cortical Aβ deposition theory (Ballard et al., 2006; Sabbagh et al., 2009; Alves et al., 2014). A recent review showed that the coexistence of amyloid and tau pathology can facilitate the process of α-synuclein aggregation, which results in an increase of insoluble fibrils representing the core of intraneuronal Lewy bodies and Lewy neurites. This process will boost a microglial reaction through inflammatory mediators, which subsequently attract peripheral immune cells within the CNS (Parnetti et al., 2019). Parallel to these pathological changes, more CSF Nfl is released.

Correlations between CSF NfL, cognitive decline, and AD-related CSF biomarkers in this study showed that these CSF biomarkers may partially share underlying processes in PD. Although anomalous CSF Aβ_42_ and tau were only detected in 6.5% of PD patients at the first diagnosis, autopsy exhibited the pathological change of AD in 60 to 80% of PD patients (Marek et al., 2018). Considered that AD type changes may contribute to PD-D, CSF Aβ42 has been shown to reliably predict the risk of cognitive impairment in PD patients (Parnetti et al., 2019), especially in verbal memory, processing speed, and visual memory (Leverenz et al., 2011; Alves et al., 2014; Yarnall et al., 2014). Therefore, A+ cut off value recognized in AD were selected to reflect the AD-related CSF profiles of enrolled patients (Shaw et al., 2018). In reference to the reputable AD cut point, approximately 1/3 of PD subjects exhibited low CSF Aβ_42_ pathologically, which indicates a positivity status of amyloid. These findings are consistent with two reported studies and one autopsy involving end-stage PD patients (Irwin et al., 2012, 2020; Ma et al., 2021). Moreover, AD patients with amyloidosis+ showed a significant association between reduced gray matter density in AD vulnerable regions and increased NfL concentrations in CSF (Kang et al., 2021). In our research, the significantly higher increase rate of NfL in A + PD-MCI, as compared to A-PD-NC was evidence that supports the assertion that dynamic CSF NfL in *de novo* PD may be consistent with AD pathological process (Olsson et al., 2019). It is also possible that amyloid deposition alone, independent of tau pathology, can hasten the development of PD cognitive impairment (Lin and Wu, 2015; Oosterveld et al., 2020). NfL was discovered in the hippocampus of PD-D patients through autopsy, but not discovered in PD-NC (Arai et al., 1992). Another explanation is that NfL leading to PD cognitive disorder is independent of Aβ pathology theory (Kovacs et al., 2017). Further research should be performed to clarify related biological and pathological mechanisms of cognitive disorder in early-stage PD.

Furthermore, CSF NfL concentration in *de novo* PD was reported to be positively correlated with age, which is consistent with studies (Bridel et al., 2019; Lerche et al., 2020; Fagan et al., 2021). There were differences in the predictive effects of NfL baseline levels/change rate on cognitive decline in PD through subgroup analyses that considered age, gender, educated years, and *APOE ε4* carrier status, respectively. Further research is required to study the interactions between these factors and CSF NfL with cognitive decline in *de novo* PD patients.

The following are the strengths of this study: (1) Enrolled PD population was newly diagnosed within a 7.4-month period as compared to more than 2 years in other studies (Lerche et al., 2020; Oosterveld et al., 2020); (2) Cross-sectional and longitudinal study designs were used; and (3) More sophisticated cognitive assessments were performed overall, rendering the prediction for conversion to dementia in PD more reasonable and reliable.

However, a few limitations of our study should be noted. First, missing values of follow-up in this longitudinal cohort inevitably influenced the reliability of our results. Second, whether there is an interaction or intermediation between NfL and Tau on the cognitive progress of PD patients remains unclear due to lack of suitable cut-off point to classify CSF tau. Third, we referenced medical history as a confounding factor and conducted sensitivity analyses, but the medication history was not enough to talk about the drug affect. Besides, quantity of CSF Aβ_42_ in our research was too little to back the transforming formula of CSF Aβ_42_ levels up with Gaussian Mixture Modelling statistics. In addition, all analyses are based on CSF protein measurements, which are not adequately accurate as compared to PET imaging data for cerebral neurodegeneration. Last but not the least, sampling of CSF NfL is an invasive procedure, and may not be obtained from all patients.

Collectively, CSF NfL can be considered as a valuable biomarker to monitor the severity of cognitive progression in *de novo* PD. Baseline levels and the change rate of CSF NfL can predict future cognitive decline in many domains. In addition, higher CSF NfL concentration tends to represent a higher risk of conversion to PD-D in *de novo* PD patients. Dynamic changes of NfL measurements may assist in identifying this risk of rapid cognitive decline for subsequent modification of therapy in future clinical trials.

## Data availability statement

The original contributions presented in the study are included in the article/Supplementary materials, further inquiries can be directed to the corresponding author.

## Ethics statement

The studies involving human participants were reviewed and approved by Parkinson’s Progression Markers Initiative. The patients/participants provided their written informed consent to participate in this study.

## Author contributions

Z-HS: design, execution, analysis, writing, and review and critique. L-ZM: design, execution, analysis, and review and critique. J-YL: design, analysis, and review and critique. Y-NO: analysis, review and critique. BZ: analysis, review and critique. Y-HM: analysis, review and critique. LT: design, analysis, review and critique, and financial support. All authors contributed to the article and approved the submitted version.

## Funding

This study was supported by grants from the National Natural Science Foundation of China (81971032, 82001133, and 81901121).

## Conflict of interest

The authors declare that the research was conducted in the absence of any commercial or financial relationships that could be construed as a potential conflict of interest.

## Publisher’s note

All claims expressed in this article are solely those of the authors and do not necessarily represent those of their affiliated organizations, or those of the publisher, the editors and the reviewers. Any product that may be evaluated in this article, or claim that may be made by its manufacturer, is not guaranteed or endorsed by the publisher.

## Footnotes

^1^http://www.ppmi-info.org, clinicaltrials.gov: NCT01141023.
