# Supplementary material for: Cerebrospinal fluid neurofilament dynamic profiles predict cognitive progression in individuals with de novo Parkinson’s disease
Source: Front Aging Neurosci. 2022 Dec 16;14:1061096. doi: 10.3389/fnagi.2022.1061096 (PMC9802677; doi:10.3389/fnagi.2022.1061096)
Supplement: Supplementary file 2 [file Data_Sheet_2.pdf]

Supplementary Material

1 Supplementary Figure 1. The Quantile-Quantile plot of NfL.

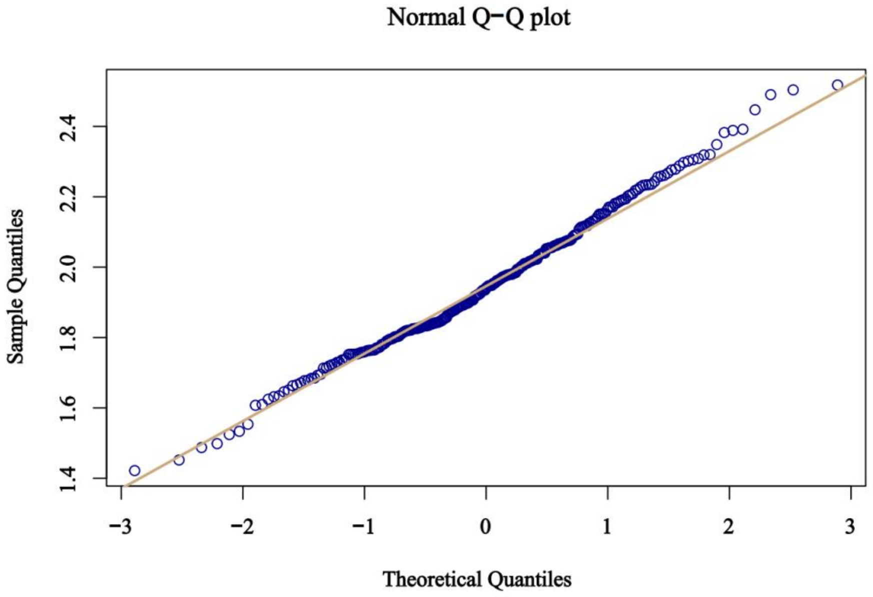

Sample quantiles were plotted follow those theoretical quantiles under the null hypothesis (x-axis). The Q-Q plot shows that log10-transformed CSF NfL data conforms to the normal distribution.

Abbreviations: CSF, cerebrospinal fluid; NfL, neurofilament light.

2 Supplementary Figure 2. CSF NfL concentration distribution with age.

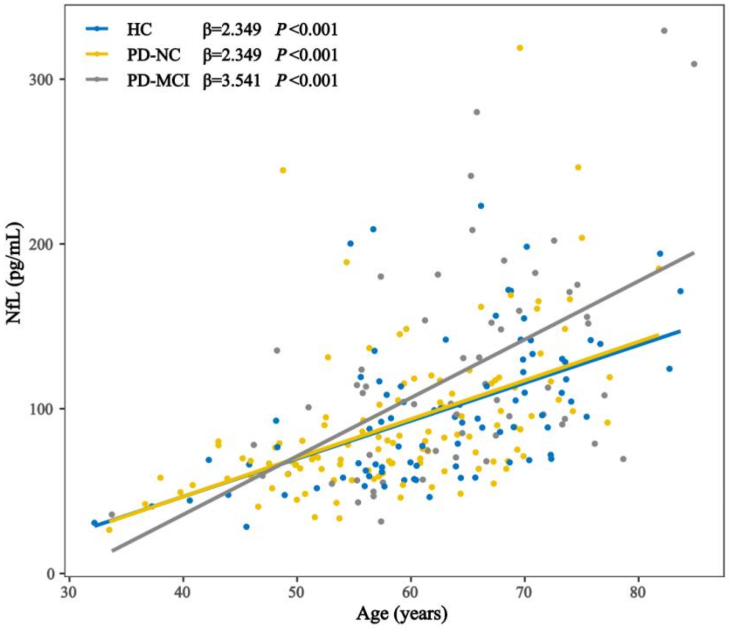

The regression coefficients (β) and *P* values computed by linear regression.

Abbreviations: CSF, cerebrospinal fluid; NfL, neurofilament light; HC, Healthy controls; PD, Parkinson's disease; PD-NC, PD patients with normal cognition; PD-MCI, PD patients with mild cognitive impairment.

3 **Supplementary Table 1. Number of data-points of longitudinal CSF biomarkers and cognitive assessments from follow-up.**

**Study visit**	**bl**	**m6**	**m12**	**m24**	**m36**	**m48**	**m60**	**m72**	**m84**	**m96**	**m108**	**m120**
$A\beta_{42}$	259	198	234	236	185	-	-	-	-	-	-	-
T-tau	259	202	237	237	216	189	169	40	77	4	5	-
P-tau	259	198	230	232	211	189	169	40	77	4	5	-
a-syn	259	202	236	237	188	-	-	-	-	-	-	-
NfL	259	174	211	201	160	161	-	-	-	-	-	-
MoCA	259	-	248	244	236	226	210	140	157	105	51	40
HVLT	259	-	246	246	235	227	210	138	159	87	15	-
BJLO	259	-	248	246	236	226	210	138	158	87	15	-
LNS	259	-	248	246	237	227	210	140	158	87	15	-
Semantic Fluency Test	259	-	248	246	237	227	210	140	158	86	15	-
SDMT	259	-	248	246	236	227	210	141	159	87	15	-

Abbreviations: CSF, Cerebrospinal fluid; $A\beta_{42}$, Amyloid-β_{42}; T-tau, Total tau; P-tau, Phosphorylated tau; α-syn, α-synuclein; NfL, Neurofilament light; MoCA, Montreal Cognitive Assessment; HVLT, Hopkins Verbal Learning Test; BJLO, Benton Judgment of Line Orientation Score; LNS, Letter Number Sequencing; SDMT, Symbol Digit Modality Test.

4 **Supplementary Table 2. Correlation of baseline NfL with CSF biomarkers and cognitive assessments observed in the HC, PD, PD-NC and PD-MCI groups.**

	HC		PD		PD-NC		PD-MCI	
**Baseline Measure**	**β**	***P* Value**	**β**	***P* Value**	**β**	***P* Value**	**β**	***P* Value**
$A\beta_{42}$	0.209	0.113	**0.182**	**0.027**	**0.224**	**0.043**	0.153	0.236
T-tau	**0.484**	**<0.001**	**0.247**	**<0.001**	**0.258**	**0.001**	**0.242**	**0.015**
P-tau	**0.540**	**<0.001**	**0.256**	**<0.001**	**0.277**	**0.001**	**0.239**	**0.028**
α-syn	**0.506**	**<0.001**	**0.192**	**0.013**	**0.230**	**0.021**	0.158	0.250
MoCA	0.007	0.592	**-0.031**	**0.045**	-0.002	0.875	-0.010	0.574
HVLT Total Recall	0.007	0.935	-0.016	0.736	0.009	0.877	-0.018	0.817
HVLT Delayed Recall	0.123	0.227	0.087	0.111	0.078	0.235	0.180	0.055
HVLT Retention	0.111	0.297	**0.139**	**0.014**	0.090	0.163	**0.269**	**0.009**
HVLT Recognition Discrimination	**0.220**	**0.047**	0.028	0.648	-0.002	0.979	0.147	0.189
BJLO	-0.095	0.304	-0.010	0.849	-0.009	0.873	0.037	0.717
LNS	-0.146	0.113	-0.030	0.572	0.029	0.618	-0.102	0.306
Semantic Fluency Test	-0.030	0.663	0.022	0.597	0.031	0.547	0.060	0.362
SDMT	-0.081	0.285	0.012	0.767	0.018	0.738	0.031	0.659

Adjusted *P* values are listed in the table.

Adjusted for age, gender, educated years, *APOE ε4* carrier status, and disease duration.

Abbreviations:CSF, Cerebrospinal fluid; NfL, Neurofilament light; HC, Healthy controls; PD, Parkinson's disease; PD-NC, PD patients with normal cognition; PD-MCI, PD patients with mild cognitive impairment; $A\beta_{42}$, Amyloid-β_{42}; T-tau, Total tau; P-tau, Phosphorylated tau; α-syn, α-synuclein; MoCA, Montreal Cognitive Assessment; HVLT, Hopkins Verbal Learning Test; BJLO, Benton Judgment of Line Orientation Score; LNS, Letter Number Sequencing; SDMT, Symbol Digit Modality Test.

Supplementary Table 3. Linear mixed-effects models for CSF biomarkers and cognitive progression in patients with *de novo* PD.

	**Baseline NfL level**			**NFL rate**		
	**Low**	**High**		**Low**	**High**	
**Measure**		**β**	***P* Value**		**β**	***P* Value**
Aβ42	Reference	0.008	0.186	Reference	-0.002	0.724
T-tau	Reference	**0.007**	**0.013**	Reference	0.004	0.118
P-tau	Reference	**0.005**	**0.037**	Reference	0.002	0.502
α-syn	Reference	0.006	0.288	Reference	0.005	0.417
MoCA	Reference	**-0.004**	**0.022**	Reference	**-0.005**	**0.001**
HVLT Total Recall	Reference	**-0.007**	**0.003**	Reference	**-0.005**	**0.027**
HVLT Delayed Recall	Reference	**-0.011**	**<0.001**	Reference	**-0.010**	**<0.001**
HVLT Retention	Reference	**-0.012**	**<0.001**	Reference	**-0.009**	**0.002**
HVLT Recognition Discrimination	Reference	**-0.008**	**0.016**	Reference	-0.004	0.187
BJLO	Reference	-0.003	0.354	Reference	-0.002	0.418
LNS	Reference	**-0.009**	**0.027**	Reference	**-0.013**	**0.002**
Semantic Fluency Test	Reference	**-0.007**	**0.002**	Reference	**-0.009**	**<0.001**
SDMT	Reference	**-0.010**	**<0.001**	Reference	**-0.013**	**<0.001**

Adjusted *P* values are listed in the table.
Adjusted for age, gender, educated years, *APOE ε4* carrier status, and disease duration.
Abbreviations: CSF, Cerebrospinal fluid; NfL, Neurofilament light; PD, Parkinson's disease; $A\beta_{42}$, Amyloid-β_{42}; T-tau, Total tau; P-tau, Phosphorylated tau; α-syn, α-synuclein; MoCA, Montreal Cognitive Assessment; HVLT, Hopkins Verbal Learning Test; BJLO, Benton Judgment of Line Orientation Score; LNS, Letter Number Sequencing; SDMT, Symbol Digit Modality Test.

6 **Supplementary Table 4. Progression risk from PD-NC or PD-MCI to PD-D.**

	**Low**	**High**	
		**HR (95% CI)**	***P* value**
**Baseline NfL level**	Reference	2.821 (1.105-7.200)	**0.030**
**NfL rate**	Reference	1.454 (0.648-3.264)	0.352

Adjusted for age, gender, educated years, *APOE ε4* carrier status, and disease duration.
Abbreviations: NfL, Neurofilament light; PD, Parkinson's disease; PD-NC, PD patients with normal cognition; PD-MCI, PD patients with mild cognitive impairment; PD-D, PD patients with dementia.

Supplementary Table 5. Linear mixed-effects models for CSF biomarkers and cognitive symptom severity in *de novo* PD patients aged <56, 56-65 and>65.

	**Baseline NfL level**						**NfL rate**					
**Age**	**<56**		**56-65**		**>65**		**<56**		**56-65**		**>65**	
**Measure**	**β**	***P* Value**	**β**	***P* Value**	**β**	***P* Value**	**β**	***P* Value**	**β**	***P* Value**	**β**	***P* Value**
$A\beta_{42}$	-0.031	0.447	0.004	0.859	-0.029	0.300	-0.0001	0.949	0.0000	0.897	-0.0006	0.027
T-tau	-0.008	0.585	**0.026**	**0.044**	0.000	0.967	0.0001	0.805	**0.0004**	**0.048**	-0.0002	0.185
P-tau	-0.009	0.514	0.018	0.099	-0.005	0.697	0.0001	0.740	0.0001	0.742	-0.0002	0.073
α-syn	-0.003	0.920	0.048	0.051	0.022	0.489	0.0006	0.480	0.0000	0.913	-0.0003	0.403
MoCA	0.002	0.549	**-0.021**	**0.030**	-0.006	0.515	0.0000	0.990	-0.0003	0.082	**-0.0002**	**0.027**
HVLT Total Recall	-0.002	0.884	-0.018	0.208	**-0.017**	**0.042**	0.0000	0.923	-0.0005	0.089	**-0.0004**	**<0.001**
HVLT Delayed Recall	-0.013	0.348	-0.027	0.096	**-0.036**	**0.004**	-0.0005	0.139	-0.0003	0.454	**-0.0008**	**<0.001**
HVLT Retention	-0.024	0.081	**-0.031**	**0.043**	**-0.041**	**0.001**	-0.0005	0.169	-0.0001	0.770	**-0.0008**	**<0.001**
HVLT Recognition Discrimination	-0.009	0.647	-0.011	0.520	0.001	0.939	0.0003	0.515	-0.0005	0.141	**-0.0003**	**0.038**
BJLO	0.002	0.887	-0.027	0.086	-0.013	0.301	0.0003	0.387	-0.0006	0.089	**-0.0004**	**0.011**
LNS	0.006	0.553	-0.029	0.167	-0.047	0.118	-0.0005	0.088	0.0004	0.380	**-0.0012**	**<0.001**
Semantic Fluency Test	-0.007	0.526	-0.021	0.070	-0.013	0.300	-0.0002	0.459	-0.0004	0.131	**-0.0006**	**<0.001**
SDMT	0.006	0.597	-0.017	0.184	**-0.026**	**0.029**	-0.0004	0.211	**-0.0007**	**0.004**	**-0.0006**	**<0.001**

Adjusted *P* values are listed in the table.

Adjusted for age, gender, educational level, *APOE ε4* carrier status, and disease duration.

Abbreviations: CSF, Cerebrospinal fluid; NfL, Neurofilament light; PD, Parkinson's disease; $A\beta_{42}$, Amyloid-β_{42}; T-tau, Total tau; P-tau, Phosphorylated tau; α-syn, α-synuclein; MoCA, Montreal Cognitive Assessment; HVLT, Hopkins Verbal Learning Test; BJLO, Benton Judgment of Line Orientation Score; LNS, Letter Number Sequencing; SDMT, Symbol Digit Modality Test.

8 **Supplementary Table 6. Linear mixed-effects models for CSF biomarkers and cognitive symptom severity in male and female patients with *de novo* PD.**

Gender	Male				Female			
	Baseline NfL level		NfL rate		Baseline NfL level		NfL rate	
Measure	β	*P* Value	β	*P* Value	β	*P* Value	β	*P* Value
$A\beta_{42}$	0.001	0.947	-0.0003	0.261	-0.020	0.413	-0.0004	0.486
T-tau	**0.022**	**0.016**	0.0000	0.859	0.005	0.621	**0.0009**	**0.005**
P-tau	**0.019**	**0.027**	-0.0001	0.591	-0.005	0.655	0.0003	0.228
α-syn	0.025	0.168	-0.0002	0.314	-0.004	0.892	0.0008	0.294
MoCA	**-0.012**	**0.050**	**-0.0002**	**0.022**	0.000	0.880	**-0.0004**	**0.003**
HVLT Total Recall	**-0.020**	**0.009**	**-0.0005**	**<0.001**	-0.008	0.456	-0.0005	0.138
HVLT Delayed Recall	**-0.032**	**0.001**	**-0.0008**	**<0.001**	**-0.025**	**0.027**	-0.0003	0.357
HVLT Retention	**-0.033**	**<0.001**	**-0.0007**	**<0.001**	**-0.035**	**0.004**	-0.0003	0.403
HVLT Recognition Discrimination	-0.017	0.112	**-0.0005**	**0.004**	-0.015	0.259	0.0004	0.296
BJLO	**-0.029**	**0.026**	**-0.0003**	**0.036**	-0.016	0.439	-0.0004	0.348
LNS	-0.008	0.397	**-0.0008**	**<0.001**	-0.013	0.267	-0.0010	0.068
Semantic Fluency Test	**-0.024**	**0.002**	**-0.0006**	**<0.001**	-0.015	0.096	**-0.0007**	**0.030**
SDMT	**-0.026**	**0.001**	**-0.0006**	**<0.001**	**-0.022**	**0.050**	**-0.0014**	**<0.001**

Adjusted *P* values are listed in the table.

Adjusted for age, gender, educated years, *APOE ε4* carrier status, and disease duration.

Abbreviations: CSF, Cerebrospinal fluid; NfL, Neurofilament light; HC, Healthy controls; PD, Parkinson's disease; PD-NC, PD patients with normal cognition; PD-MCI, PD patients with mild cognitive impairment; $A\beta_{42}$, Amyloid-β_{42}; T-tau, Total tau; P-tau, Phosphorylated tau; α-syn, α-synuclein; MoCA, Montreal Cognitive Assessment; HVLT, Hopkins Verbal Learning Test; BJLO, Benton Judgment of Line Orientation Score; LNS, Letter Number Sequencing; SDMT, Symbol Digit Modality Test.

9 **Supplementary Table 7. Linear mixed-effects models for CSF biomarkers and cognitive symptom severity in *de novo* PD patients with educated years ≥13 and <13.**

	≥13				<13			
	Baseline NfL level		NfL rate		Baseline NfL level		NfL rate	
**Measure**	**β**	***P* Value**	**β**	***P* Value**	**β**	***P* Value**	**β**	***P* Value**
$A\beta_{42}$	-0.091	0.316	-0.0014	0.457	0.004	0.761	-0.0002	0.240
T-tau	0.038	0.186	0.0011	0.056	0.012	0.075	0.0001	0.566
P-tau	**0.052**	**0.043**	-0.0001	0.439	0.006	0.371	**0.0013**	**0.013**
α-syn	-0.005	0.906	0.0004	0.663	0.020	0.172	-0.0001	0.547
MoCA	-0.004	0.656	0.0000	0.959	**-0.011**	**0.011**	**-0.0003**	**0.001**
HVLT Total Recall	-0.016	0.506	-0.0006	0.318	**-0.016**	**0.006**	**-0.0005**	**<0.001**
HVLT Delayed Recall	-0.047	0.180	**-0.0018**	**0.015**	**-0.028**	**<0.001**	**-0.0007**	**<0.001**
HVLT Retention	**-0.059**	**0.031**	**-0.0018**	**0.008**	**-0.031**	**<0.001**	**-0.0006**	**<0.001**
HVLT Recognition Discrimination	-0.005	0.867	-0.0001	0.929	-0.016	0.059	**-0.0004**	**0.012**
BJLO	-0.027	0.117	0.0000	0.964	-0.010	0.201	**-0.0004**	**0.011**
LNS	-0.004	0.819	-0.0001	0.782	**-0.029**	**0.009**	**-0.0008**	**<0.001**
Semantic Fluency Test	-0.024	0.167	-0.0005	0.179	**-0.022**	**<0.001**	**-0.0006**	**<0.001**
SDMT	-0.039	0.085	-0.0007	0.162	**-0.023**	**<0.001**	**-0.0007**	**<0.001**

Adjusted *P* values are listed in the table.
Adjusted for age, gender, educated years, *APOE ε4* carrier status, and disease duration.
Abbreviations: CSF, Cerebrospinal fluid; NfL, Neurofilament light; HC, Healthy controls; PD, Parkinson's disease; PD-NC, PD patients with normal cognition; PD-MCI, PD patients with mild cognitive impairment; $A\beta_{42}$, Amyloid-β_{42}; T-tau, Total tau; P-tau, Phosphorylated tau; α-syn, α-synuclein; MoCA, Montreal Cognitive Assessment; HVLT, Hopkins Verbal Learning Test; BJLO, Benton Judgment of Line Orientation Score; LNS, Letter Number Sequencing; SDMT, Symbol Digit Modality Test.

Supplementary Table 8. Linear mixed-effects models for CSF biomarkers and cognitive symptom severity in *de novo* PD patients carrying *APOE ε4* or not.

***APOE ε4* carrier status**	**Carried**				**Not carried**			
	**Baseline NfL level**		**NfL rate**		**Baseline NfL level**		**NfL rate**	
**Measure**	**β**	***P* Value**	**β**	***P* Value**	**β**	***P* Value**	**β**	***P* Value**
Aβ_{42}	-0.017	0.525	-0.0003	0.204	0.005	0.759	0.0000	0.921
T-tau	0.009	0.520	-0.0001	0.442	**0.016**	**0.046**	**0.0006**	**0.005**
P-tau	0.006	0.691	-0.0002	0.108	0.011	0.139	**0.0004**	**0.033**
α-syn	-0.016	0.448	**-0.0004**	**0.028**	0.031	0.083	0.0004	0.250
MoCA	-0.018	0.108	**-0.0003**	**0.033**	**-0.008**	**0.049**	-0.0002	0.120
HVLT Total Recall	-0.020	0.181	**-0.0006**	**0.001**	**-0.015**	**0.013**	-0.0003	0.078
HVLT Delayed Recall	-0.028	0.095	**-0.0008**	**<0.001**	**-0.031**	**<0.001**	**-0.0007**	**0.001**
HVLT Retention	-0.024	0.124	**-0.0006**	**0.002**	**-0.037**	**<0.001**	**-0.0007**	**0.001**
HVLT Recognition Discrimination	-0.007	0.727	**-0.0005**	**0.025**	-0.018	0.051	-0.0002	0.438
BJLO	-0.024	0.150	**-0.0006**	**0.004**	-0.006	0.452	0.0001	0.612
LNS	-0.021	0.360	**-0.0009**	**<0.001**	**-0.029**	**0.009**	**-0.0008**	**0.006**
Semantic Fluency Test	**-0.039**	**0.005**	**-0.0007**	**<0.001**	**-0.017**	**0.010**	**-0.0005**	**0.004**
SDMT	**-0.032**	**0.040**	**-0.0008**	**<0.001**	**-0.022**	**<0.001**	**-0.0006**	**<0.001**

Adjusted *P* values are listed in the table.

Adjusted for age, gender, educated years, *APOE ε4* carrier status, and disease duration.

Abbreviations: CSF, Cerebrospinal fluid; NfL, Neurofilament light; HC, Healthy controls; PD, Parkinson's disease; PD-NC, PD patients with normal cognition; PD-MCI, PD patients with mild cognitive impairment; Aβ_{42}, Amyloid-β_{42}; T-tau, Total tau; P-tau, Phosphorylated tau; α-syn, α-synuclein; MoCA, Montreal Cognitive Assessment; HVLT, Hopkins Verbal Learning Test; BJLO, Benton Judgment of Line Orientation Score; LNS, Letter Number Sequencing; SDMT, Symbol Digit Modality Test.

11 **Supplementary Table 9. Linear mixed-effects models for CSF biomarkers and cognitive symptom severity in patients with PD-NC and PD-MCI.**

**Cognitive diagnosis**	**PD-NC**				**PD-MCI**			
	**Baseline NfL level**		**NfL rate**		**Baseline NfL level**		**NfL rate**	
**Measure**	**β**	***P* Value**	**β**	***P* Value**	**β**	***P* Value**	**β**	***P* Value**
$A\beta_{42}$	0.0097	0.633	0.0003	0.572	-0.0276	0.198	**-0.0005**	**0.028**
T-tau	0.0107	0.225	0.0003	0.287	0.0154	0.183	0.0000	0.829
P-tau	0.0070	0.380	0.0002	0.519	0.0081	0.464	-0.0001	0.373
α-syn	0.0147	0.426	0.0003	0.580	0.0228	0.373	-0.0002	0.426
MoCA	**-0.0058**	**0.041**	-0.0001	0.098	-0.0136	0.223	-0.0003	0.070
HVLT Total Recall	-0.0119	0.122	**-0.0006**	**0.006**	**-0.0216**	**0.022**	**-0.0004**	**0.004**
HVLT Delayed Recall	**-0.0202**	**0.021**	**-0.0006**	**0.012**	**-0.0453**	**<0.001**	**-0.0008**	**<0.001**
HVLT Retention	**-0.0248**	**0.005**	-0.0004	0.170	**-0.0470**	**<0.001**	**-0.0008**	**<0.001**
HVLT Recognition Discrimination	-0.0053	0.601	-0.0003	0.278	-0.0281	0.061	-0.0004	0.084
BJLO	0.0033	0.690	-0.0002	0.384	**-0.0320**	**0.018**	-0.0004	0.055
LNS	**-0.0160**	**0.035**	**-0.0007**	**0.001**	-0.0333	0.252	-0.0007	0.051
Semantic Fluency Test	-0.0125	0.101	**-0.0006**	**0.007**	**-0.0356**	**<0.001**	**-0.0006**	**<0.001**
SDMT	-0.0125	0.086	**-0.0009**	**<0.001**	**-0.0347**	**0.001**	**-0.0005**	**<0.001**

Adjusted *P* values are listed in the table.
Adjusted for age, gender, educated years, *APOE ε4* carrier status, and disease duration.
Abbreviations: CSF, Cerebrospinal fluid; NfL, Neurofilament light; HC, Healthy controls; PD, Parkinson's disease; PD-NC, PD patients with normal cognition; PD-MCI, PD patients with mild cognitive impairment; $A\beta_{42}$, Amyloid-β_{42}; T-tau, Total tau; P-tau, Phosphorylated tau; α-syn, α-synuclein; MoCA, Montreal Cognitive Assessment; HVLT, Hopkins Verbal Learning Test; BJLO, Benton Judgment of Line Orientation Score; LNS, Letter Number Sequencing; SDMT, Symbol Digit Modality Test.

12 **Supplementary Table 10. Linear mixed-effects models for CSF biomarkers and cognitive symptom severity in patients with Amyloid- PD and Amyloid+ PD.**

**Amyloid status**	**Amyloid- PD**				**Amyloid+ PD**			
	**Baseline NfL level**		**NfL rate**		**Baseline NfL level**		**NfL rate**	
**Measure**	**β**	***P* Value**	**β**	***P* Value**	**β**	***P* Value**	**β**	***P* Value**
$A\beta_{42}$	0.0225	0.143	0.0002	0.523	-0.0576	0.072	**-0.0007**	**0.029**
T-tau	**0.0159**	**0.043**	**0.0005**	**0.009**	0.0148	0.306	-0.0001	0.683
P-tau	**0.0139**	**0.047**	**0.0004**	**0.014**	0.0014	0.918	-0.0002	0.119
α-syn	0.0204	0.235	0.0005	0.234	0.0171	0.496	-0.0003	0.213
MoCA	-0.0064	0.085	-0.0001	0.250	**-0.0262**	**0.035**	**-0.0003**	**0.031**
HVLT Total Recall	**-0.0182**	**0.002**	-0.0003	0.069	-0.0141	0.344	**-0.0005**	**0.003**
HVLT Delayed Recall	**-0.0325**	**<0.001**	**-0.0007**	**<0.001**	-0.0250	0.126	**-0.0007**	**<0.001**
HVLT Retention	**-0.0355**	**<0.001**	**-0.0007**	**0.001**	-0.0288	0.057	**-0.0006**	**0.002**
HVLT Recognition Discrimination	**-0.0222**	**0.013**	**-0.0005**	**0.022**	0.0087	0.651	-0.0002	0.312
BJLO	-0.0083	0.300	0.0001	0.620	-0.0246	0.091	**-0.0006**	**0.001**
LNS	-0.0096	0.288	-0.0001	0.645	**-0.0794**	**0.008**	**-0.0011**	**0.001**
Semantic Fluency Test	**-0.0140**	**0.030**	**-0.0004**	**0.009**	**-0.0455**	**0.001**	**-0.0007**	**<0.001**
SDMT	**-0.0201**	**0.001**	**-0.0007**	**<0.001**	**-0.0408**	**0.009**	**-0.0006**	**<0.001**

Adjusted *P* values are listed in the table.
Adjusted for age, gender, educated years, *APOE ε4* carrier status, and disease duration.
Abbreviations: CSF, Cerebrospinal fluid; NfL, Neurofilament light; HC, Healthy controls; PD, Parkinson's disease; PD-NC, PD patients with normal cognition; PD-MCI, PD patients with mild cognitive impairment; $A\beta_{42}$, Amyloid-β_{42}; T-tau, Total tau; P-tau, Phosphorylated tau; α-syn, α-synuclein; MoCA, Montreal Cognitive Assessment; HVLT, Hopkins Verbal Learning Test; BJLO, Benton Judgment of Line Orientation Score; LNS, Letter Number Sequencing; SDMT, Symbol Digit Modality Test.

13 **Supplementary Table 11. Correlation of baseline NfL with CSF biomarkers and cognitive assessments observed in the HC, PD, PD-NC and PD-MCI groups after adjusting for medical comorbidities.**

	HC		PD		PD-NC		PD-MCI	
**Baseline Measure**	**β**	***P* Value**	**β**	***P* Value**	**β**	***P* Value**	**β**	***P* Value**
$A\beta_{42}$	0.212	0.114	**0.192**	**0.020**	**0.238**	**0.031**	0.152	0.246
T-tau	**0.471**	**<0.001**	**0.240**	**<0.001**	**0.254**	**0.001**	**0.229**	**0.021**
P-tau	**0.519**	**<0.001**	**0.247**	**<0.001**	**0.270**	**0.001**	**0.228**	**0.037**
α-syn	**0.506**	**<0.001**	**0.186**	**0.017**	**0.225**	**0.024**	0.146	0.296
MoCA	0.003	0.822	**-0.033**	**0.032**	-0.002	0.812	-0.015	0.402
HVLT Total Recall	0.014	0.869	-0.016	0.735	0.008	0.898	-0.011	0.895
HVLT Delayed Recall	0.111	0.283	0.085	0.124	0.072	0.275	**0.189**	**0.046**
HVLT Retention	0.089	0.405	**0.135**	**0.018**	0.082	0.201	**0.275**	**0.009**
HVLT Recognition Discrimination	0.182	0.094	0.022	0.719	-0.009	0.902	0.147	0.199
BJLO	-0.067	0.464	-0.018	0.725	-0.013	0.812	0.024	0.814
LNS	-0.159	0.090	-0.033	0.535	0.030	0.613	-0.115	0.256
Semantic Fluency Test	-0.021	0.763	0.020	0.639	0.030	0.557	0.058	0.389
SDMT	-0.082	0.287	0.015	0.727	0.022	0.686	0.026	0.709

Adjusted *P* values are listed in the table.
Adjusted for age, gender, educated years, *APOE ε4* carrier status, disease duration and medical comorbidities.
Abbreviations:CSF, Cerebrospinal fluid; NfL, Neurofilament light; HC, Healthy controls; PD, Parkinson's disease; PD-NC, PD patients with normal cognition; PD-MCI, PD patients with mild cognitive impairment; $A\beta_{42}$, Amyloid-β_{42}; T-tau, Total tau; P-tau, Phosphorylated tau; α-syn, α-synuclein; MoCA, Montreal Cognitive Assessment; HVLT, Hopkins Verbal Learning Test; BJLO, Benton Judgment of Line Orientation Score; LNS, Letter Number Sequencing; SDMT, Symbol Digit Modality Test.

14 **Supplementary Table 12. Linear mixed-effects models for CSF biomarkers and cognitive progression in patients with *de novo* PD after adjusting for medical comorbidities.**

	Baseline NfL level		NFL rate	
**Measure**	**β**	***P* Value**	**β**	***P* Value**
$A\beta_{42}$	-0.0012	0.934	-0.0003	0.232
T-tau	**0.0144**	**0.037**	0.0001	0.285
P-tau	0.0094	0.136	0.0000	0.944
α-syn	0.0190	0.174	-0.0001	0.674
MoCA	**-0.0103**	**0.011**	**-0.0002**	**0.001**
HVLT Total Recall	**-0.0161**	**0.006**	**-0.0005**	**<0.001**
HVLT Delayed Recall	**-0.0297**	**<0.001**	**-0.0007**	**<0.001**
HVLT Retention	**-0.0335**	**<0.001**	**-0.0006**	**<0.001**
HVLT Recognition Discrimination	-0.0141	0.085	**-0.0004**	**0.018**
BJLO	-0.0116	0.105	**-0.0003**	**0.015**
LNS	**-0.0265**	**0.008**	**-0.0008**	**<0.001**
Semantic Fluency Test	**-0.0225**	**<0.001**	**-0.0006**	**<0.001**
SDMT	**-0.0246**	**<0.001**	**-0.0007**	**<0.001**

Adjusted *P* values are listed in the table.
Adjusted for age, gender, educated years, *APOE ε4* carrier status, disease duration and medical comorbidities.
Abbreviations: CSF, Cerebrospinal fluid; NfL, Neurofilament light; PD, Parkinson’s disease; $A\beta_{42}$, Amyloid-β_{42}; T-tau, Total tau; P-tau, Phosphorylated tau; α-syn, α-synuclein; MoCA, Montreal Cognitive Assessment; HVLT, Hopkins Verbal Learning Test; BJLO, Benton Judgment of Line Orientation Score; LNS, Letter Number Sequencing; SDMT, Symbol Digit Modality Test.

15 **Supplementary Table 13. Progression risk from PD-NC or PD-MCI to PD-D after adjusting for medical comorbidities.**

	**Low**	**High**	
		**HR (95% CI)**	***P* value**
**Baseline NfL level**	Reference	2.888 (1.122-7.435)	**0.028**
**NfL rate**	Reference	1.449 (0.645-3.255)	0.370

Adjusted for age, gender, educated years, *APOE ε4* carrier status, disease duration and medical comorbidities.

Abbreviations: NfL, Neurofilament light; PD, Parkinson's disease; PD-NC, PD patients with normal cognition; PD-MCI, PD patients with mild cognitive impairment; PD-D, PD patients with dementia.
